# Comparative genotypic characterization related to antibiotic resistance phenotypes of clinical carbapenem-resistant *Acinetobacter baumannii* MTC1106 (ST2) and MTC0619 (ST25)

**DOI:** 10.1186/s12864-023-09734-2

**Published:** 2023-11-17

**Authors:** Made Rai Dwitya Wiradiputra, Krit Thirapanmethee, Piyatip Khuntayaporn, Pagakrong Wanapaisan, Mullika Traidej Chomnawang

**Affiliations:** 1 Antimicrobial Resistance Interdisciplinary Group (AmRIG), Faculty of Pharmacy, Mahidol University, Bangkok, Thailand; 2 Biopharmaceutical Sciences Program, Faculty of Pharmacy, Mahidol University, Bangkok, Thailand; 3 Department of Microbiology, Faculty of Pharmacy, Mahidol University, Bangkok, Thailand

**Keywords:** *A. baumannii*, Antimicrobial resistance, Carbapenem, CRAB, ST2, ST25

## Abstract

**Background:**

The prevalence of *Acinetobacter baumannii* in nosocomial infections and its remarkable ability to develop antimicrobial resistance have been a critical issue in hospital settings. Here, we examined the genomic features related to resistance phenotype displayed by carbapenem-resistant *A. baumannii* (CRAB) MTC1106 (ST2) and MTC0619 (ST25).

**Results:**

Resistome analysis of both strains revealed that MTC1106 possessed higher numbers of antimicrobial resistance genes compared to MTC0619. Some of those genetic determinants were present in accordance with the susceptibility profile of the isolates. The predicted ISAba1 region upstream of *bla*_*OXA-23*_ gene was related to carbapenem resistance since this IS element was well-characterized to mediate overexpression of carbapenemase genes and eventually provided capability to confer resistance. Unlike MTC0619 strain, which only carried class B and D β-lactamase genes, MTC1106 strain also possessed *bla*_*TEM-1D*_, a class A β-lactamase. Regarding to aminoglycosides resistance, MTC0619 contained 5 related genes in which all of them belonged to three groups of aminoglycosides modifying enzyme (AME), namely, N-acetyltransferase (AAC), O-nucleotidyltransferase (ANT), and O-phosphotransferase (APH). On the other hand, MTC1106 lacked only the AAC of which found in MTC0619, yet it also carried an *armA* gene encoding for 16S rRNA methyltransferase. Two macrolides resistance genes, *mph(E)* and *msr(E)*, were identified next to the *armA* gene of MTC1106 isolate in which they encoded for macrolide 2’-phosphotransferase and ABC-type efflux pump, respectively. Besides acquired resistance genes, some chromosomal genes and SNPs associated with resistance to fluoroquinolones (i.e. *gyrA* and *parC*) and colistin (i.e. *pmrCAB*, *eptA*, and *emrAB*) were observed. However, gene expression analysis suggested that the genetic determinants significantly contributing to low-level colistin resistance remained unclear. In addition, similar number of efflux pumps genes were identified in both lineages with only the absence of *adeC*, a part of *adeABC* RND-type multidrug efflux pump in MTC0619 strain.

**Conclusions:**

We found that MTC1106 strain harbored more antimicrobial resistance genes and showed higher resistance to antibiotics than MTC0619 strain. Regarding genomic characterization, this study was likely the first genome comparative analysis of CARB that specifically included isolates belonging to ST2 and ST25 which were widely spread in Thailand. Taken altogether, this study suggests the importance to monitor the resistance status of circulating *A. baumannii* clones and identify genes that may contribute to shifting the resistance trend among isolates.

**Supplementary Information:**

The online version contains supplementary material available at 10.1186/s12864-023-09734-2.

## Background

*Acinetobacter baumannii* is the most common member of ESKAPE organisms (*Enterococcus faecium*, *Staphylococcus aureus*, *Klebsiella pneumoniae*, *Acinetobacter baumannii*, *Pseudomonas aeruginosa*, and *Enterobacter* species), a prominent group of bacterial pathogens that primarily contribute to nosocomial infections and exhibit a concerning prevalence of antimicrobial resistance (AMR) [1]. The development of resistance in ESKAPE organisms and other pathogens has been recognized as one of the major threats with multifaceted detrimental consequences on human health. Addressing this issue necessitates collaborative efforts across various disciplines [2, 3]. Moreover, the rapid emergence of resistance in some of those bacteria may give rise to “superbugs” that are resistant to most available antimicrobials, potentially resulting a crisis regarding the availability of effective treatment options. Responding to the escalating trend of resistance and the need for new antimicrobials, the World Health Organization (WHO) has included ESKAPE organisms in the global priority pathogen list, with carbapenem-resistant *A. baumannii* (CRAB) being among the most critical concerns [4].

Carbapenems are the mainstay antibiotics for the treatment of infections caused by multidrug-resistant (MDR) *A. baumannii*. However, the increasing resistance rates to these antimicrobials has become a public health concern as such strains are getting more frequently observed to cause difficult-to-treat infections and hospital outbreaks [5]. According to the most recent Global Antimicrobial Resistance and Use Surveillance System (GLASS) report in 2021, *A. baumannii* was prevalent among patients with blood stream infection (BSI) in 68 countries, with over 60% of the reported isolates demonstrating resistance to carbapenems [6]. It is also worth noting that CRAB isolates often display a MDR phenotype, and some are evolving toward an extensively drug-resistant (XDR) phenotype. This evolution severely limits treatment options and potentially leading to untreatable infections [7–9]. Resistance to carbapenems also implicates the crtitical importance of preserving colistin as one of the last-line antimicrobials to treat CRAB-associated infections. However, colistin-resistant strains with XDR or pan drug-resistant (PDR) phenotype have been reported in various regions [10–12].

The development of AMR in *A. baumannii* is associated with its genome plasticity, which allows the acquisition of functional genetic determinants such as antimicrobial resistance and virulence genes that enhance its survival. The genomic characteristics of *A. baumannii* have become an intriguing area of research in understanding the epidemiology and evolutionary trajectories of this pathogen. Several studies investigating the genomic features of clinical isolates have indicated that *A. baumannii* possesses a large proportion of accessory genome comprising flexible gene pools that rapidly evolve through environmental selective pressure, resulting in the dynamic genome reorganization and intra-clonal diversity [13, 14]. On the other hand, the core genome itself is relatively homogenous among closely related strains. Studies on the diversity and population structure of *A. baumannii* have revealed that numerous MDR strains isolated from different regions around the world were spread through clonal expansion and may share common ancestors with epidemic lineages initially identified during hospital outbreaks in Europe. The global dissemination of this organism is predicted to contribute to the emergence of resistance to multiple antimicrobial agents, including carbapenems, as this event can introduce various ecological niches that facilitates the selection of resistant clones. To date, three major clonal lineages have been globally distributed and at least five others have had limited dissemination in specific regions [15, 16].

Elucidating the resistance mechanism is another integral part of AMR surveillance and notably an important aspect in developing a novel strategy against drug-resistant bacteria. In *A. baumannii*, the presence of several intrinsic and acquired genetic determinants has been linked to multiple resistance traits. The extent to which these determinants contribute to drug resistance varies, depending on their activity and the nature of the genetic locus in which the corresponding AMR genes are found. For instance, the naturally occurring AmpC β-lactamase (*bla*_*ADC*_) can confer resistance to extended-spectrum cephalosporins when its expression is enhanced by the presence of the insertion sequence ISAba1 located upstream of the gene. In contrast, the basal expression of this gene alone is insufficient to induce resistance [17, 18]. In terms of carbapenem resistance, the mechanism in *A. baumannii* is primarily mediated by carbapenem-hydrolyzing class D β-lactamases (CHDLs) and the presence of insertion sequence (IS) elements is frequently associated with clinical significance [19]. However, a single CHDL gene may not be adequate to confer resistance although it carries the IS element. This exemplified by the presence of intrinsic OXA-51 family serine-type oxacillinase (*bla*_*OXA-51-like*_) associated with ISAba1 in some carbapenem-susceptible isolates. In the presence of IS elements, the difference in carbapenem hydrolyzing activity between *bla*_*OXA-51-like*_ alleles and amino acid substitution in the active site of OXA-51 were shown to underly its propensity to confer carbapenem-resistant phenotype [20]. Despite the prominence of acquired β-lactamases, several chromosomal genes, including efflux pumps and membrane porins, have been linked to carbapenem resistance in *A. baumannii* [21, 22]. It underlines the idea that resistance traits may not necessarily be caused by a single AMR gene but rather involve a complex mechanism due to interplay between multiple genetic determinants. Therefore, the genomic characterization of drug-resistant *A. baumannii* is plausible to provide a comprehensive insight into its genetic makeup and elucidate the intricate association with the resistant phenotype. In this present work, a comparative genomic analysis was carried out to identify genetic determinants related to AMR between carbapenem-resistant *A. baumannii* ST2 and ST25 lineages. The data is also feasible for a deeper understanding of resistance mechanisms and may eventually lead to the identification of molecular targets that can be considered in developing strategies against AMR.

## Results

### Genomic characteristics of clinical carbapenem-resistant *Acinetobacter baumannii* ST2 and ST25 lineages

Previous studies have highlighted that most CRAB isolates in Thailand were of ST2 lineage, but some belonged to ST25 and other types [9, 23, 24]. Here, we characterized the genomic diversity of two CRAB strains MTC1106 (ST2) and MTC0619 (ST25) spreading in tertiary hospitals in Thailand. The de novo assembly of the filtered raw reads from MTC0619 resulted 62 contigs, with an N50 length of 155,743 bp and a total genome size of 4,158,127 bp (Table 1). The longest and shortest contigs were 344,197 bp and 580 bp, respectively. Meanwhile, the assembly of MTC1106 generated 73 contigs with an N50 length of 122,336 bp and a total genome size of 3,922,423 bp. The longest and shortest contigs were 245,764 bp and 505 bp, respectively. Although the draft genome size for MTC0619 was slightly larger than that of MTC1106, both isolates exhibited nearly identical GC content (38.88% for MTC0619 and 38.94% for MTC1106). The size and GC content of the draft genomes from this study fell within the range observed in publicly available *A. baumannii* complete genomes deposited in the NCBI genome assembly database (https://www.ncbi.nlm.nih.gov/genome/browse/). As of September, 2023, the NCBI database contained 579 complete genomes of *A. baumannii* with the sizes ranging from 3.63 Mb to 4.57 Mb and GC content ranging between 38.76–39.70%. All reads were mapped to the contigs and no base mismatches were identified. The estimation of genome completeness using CheckM, with *A. baumannii* as the marker lineage, indicated that only a small proportion of the lineage-specific marker genes absent in both isolates. Based on the annotation of the draft genome using Prokka program, MTC0619 was predicted to have 3,918 genes, while MTC1106 was predicted to have 3,674 genes. On the other hand, the RASTtk pipeline indicated 4,018 genes in MTC0619 and 3,842 genes in MTC1106.

**Table 1:** General characteristics of MTC0619 and MTC1106 genomes

Feature	MTC0619 (ST25)	MTC1106 (ST2)
ST (Pasteur)	25	2
ST (Oxford)	229	195/1816
KL type	KL14	KL3
OCL type	OCL6	OCL1
Predicted CDS		
– Prokka	3,918	3,674
– RASTtk	4,018	3,842
tRNAs	64	63
rRNAs	5	4
Hypothetical proteins		
– Prokka	1,701	1,571
– RASTtk	1,167	912
Genes assigned to COG	3,244	3,080
Genes assigned to KEGG orthology	2,095	2,005
Predicted IS elements		
– MobileElementFinder	6	3
– ISEScan	20	12
Predicted virulence genes (VFDB)	75	78
Predicted resistance genes		
– NCBI AMRFinder	10	12
– ResFinder	9	11
– MEGARes	30	33
– CARD	26	29
Predicted prophage region (PHASTER)		
– Intact	2	0
– Questionable	1	0
– Incomplete	4	7

### In silico molecular typing

Molecular typing using both Pasteur and Oxford schemes MLST identified all seven housekeeping gene alleles with 100% coverage and identity in each respective scheme. The Pasteur MLST scheme successfully confirmed that that MTC0619 and MTC1106 belonged to ST25 and ST2, respectively. In the Oxford scheme MLST, MTC0619 was assigned to ST229. Interestingly, MTC1106 was classified into two different STs (195/1816) in the Oxford scheme owing to the presence of two *gdhB* alleles (*gdhB_3* and *gdhB_189*) within its genome.

Additionally, molecular typing based on genetic loci involved in biosynthesis of capsular polysaccharides (K locus) and outer core of lipopolysaccharides (OC locus) using Kaptive database revealed that MTC0619 belonged to KL14 and OCL6, while MTC1106 belonged to KL3 and OCL1 with at least “very high” match confidence category. A linear comparison of the K locus between isolates in the present study and five *A. baumannii* reference strains showed that the arrangement of the genes constituting this locus was identical between MTC1106, the reference strain ATCC19606, and ATCC17978 (Fig. S1A). In the OC locus, three reference strains (i.e., ACICU, AYE, and ATCC19606) exhibited identical loci to that of MTC1106. Conversely, none of the reference strains possessed KL or OCL types similar to MTC0619 (Fig. S1B).

### Resistome analysis related to antimicrobial resistance phenotype

The overall antimicrobial susceptibility profile of these CRAB isolates to sixteen antimicrobial agents revealed 100% resistance rates to aminoglycosides, carbapenems, cephalosporins, piperacillin/tazobactam, trimethoprim/sulfamethoxazole, and colistin (Table 2). Notably, both isolates remained susceptible to tigecycline. MTC0619 displayed a distinctive susceptibility pattern, as it was sensitive to levofloxacin and two other categories of antimicrobials, namely penicillins with β-lactamase inhibitors and tetracyclines.

**Table 2:**
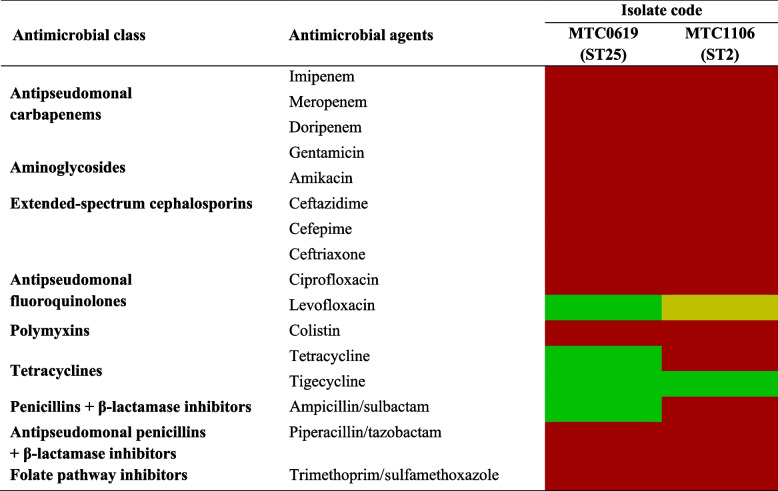
Antibiogram of carbapenem-resistant *A. baumannii* MTC1106 and MTC0619

Resistant

Intermediate

Susceptible

^a^Susceptibility was based on CLSI clinical breakpoints for *Acinetobacter* spp., except the tigecycline

**Fig. 1:**
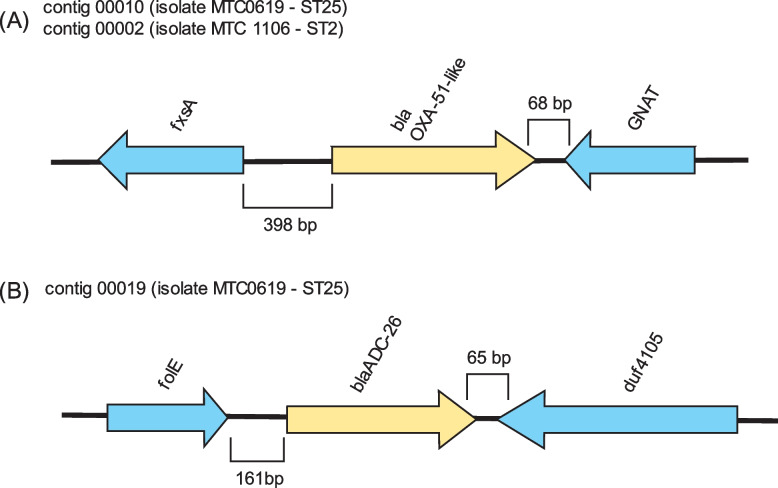
Schematic illustration of genetic structure of *bla*_OXA-51-like_ (**A**) and *bla*_ADC-26_ (**B**). The β-lactamase genes are shown in yellow arrows while any characterized genes surrounding them are marked as blue arrows

**Fig. 2:**
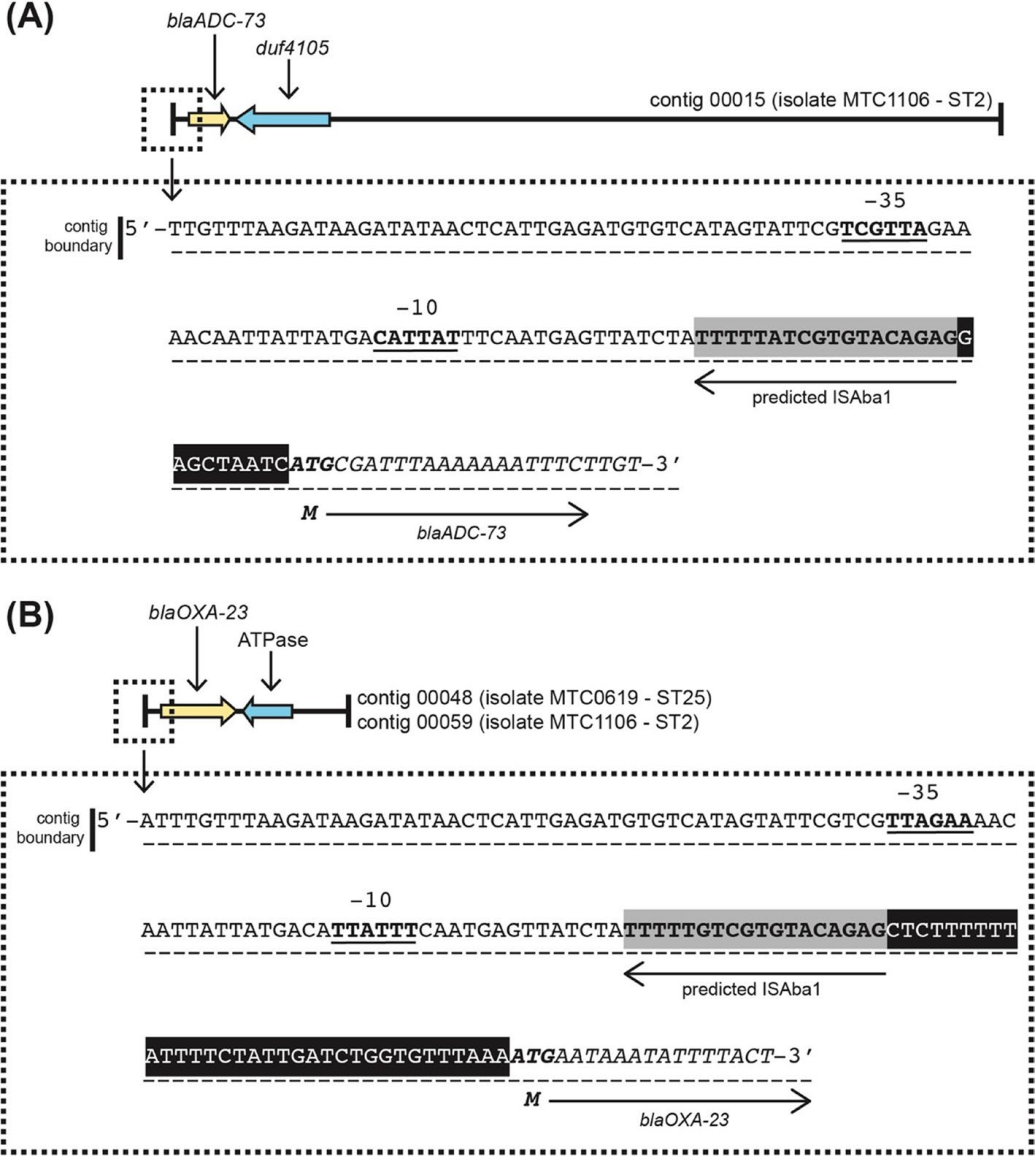
The partial sequence of ISAba1 in the upstream *bla*_*ADC-73*_ gene of MTC0619 (**A**) and *bla*_*OXA-23*_ of MTC1106 and MTC0619 (**B**). The putative -35 and -10 regions of the promoter are in bold and underlined text. The left inverted repeat (IRL) of ISAba1 is marked in grey shade followed by the direct repeat sequence shown as black-shaded white text. The *bla*_*ADC-73*_ or *bla*_*OXA-23*_ sequences are in italic with the start codon (M) shown in bold italic

Screening for the presence of AMR associated genes using ResFinder [25], NCBI AMRFinder Plus [26], CARD [27], and MEGARes [28] databases identified some resistance genes encoding efflux pumps and outer membrane proteins (Table 3). Moreover, several resistance-associated chromosomal mutations were discovered by aligning the corresponding genes between the isolates in this study with other *A. baumannii* reference strains (ATCC 17978 and SDF). Compared to MTC0619, the draft genome of isolate MTC1106 appeared to carry more AMR genes which were related to resistance against eight groups of antimicrobials. Both isolates had the intrinsic β-lactamases *bla*_*OXA-51-like*_ and *bla*_*ADC*_ genes. In the case of *bla*_*OXA-51-like*_, MTC0619 harbored *bla*_*OXA-64*_ allele in contig 00010, while MTC1106 carried *bla*_*OXA-66*_ allele in contig 00002. Notably, no IS elements were found adjacent to the OXA-51-like genes (Fig. 1). Instead, they were flanked by a gene encoding N-acetyltransferase with an intergenic spacer of 68 bp in the upstream region and *fsxA* gene (a putative suppressor of F exclusion of phage T7) with an intergenic spacer of 398 bp in the downstream region. The *bla*_*ADC-26*_ allele was identified in MTC0619 without any associated IS elements. In the case of MTC1106, the *bla*_*ADC-73*_ allele was initially identified without an IS element. However, a more detailed analysis of the nucleotide sequence surrounding this gene revealed the presence of a truncated IS element in the upstream region. This IS element was predicted to belong to ISAba1 since the left inverted repeat sequence of this mobile element was observed in the upstream region, connected to the *bla*_*ADC-73*_ gene by a 9-bp direct repeat (Fig. 2). A similar situation also observed during sequence analysis of the *bla*_*OXA-23*_ gene. Both MTC0619 and MTC1106 carried a single *bla*_*OXA-23*_ gene with partial sequence of ISAba1 and its associated inverted repeat in the upstream region. The *bla*_*TEM-1*D_, which belongs to the class A β-lactamase, was specifically present in MTC0619 but absent in MTC1106.

**Table 3:**
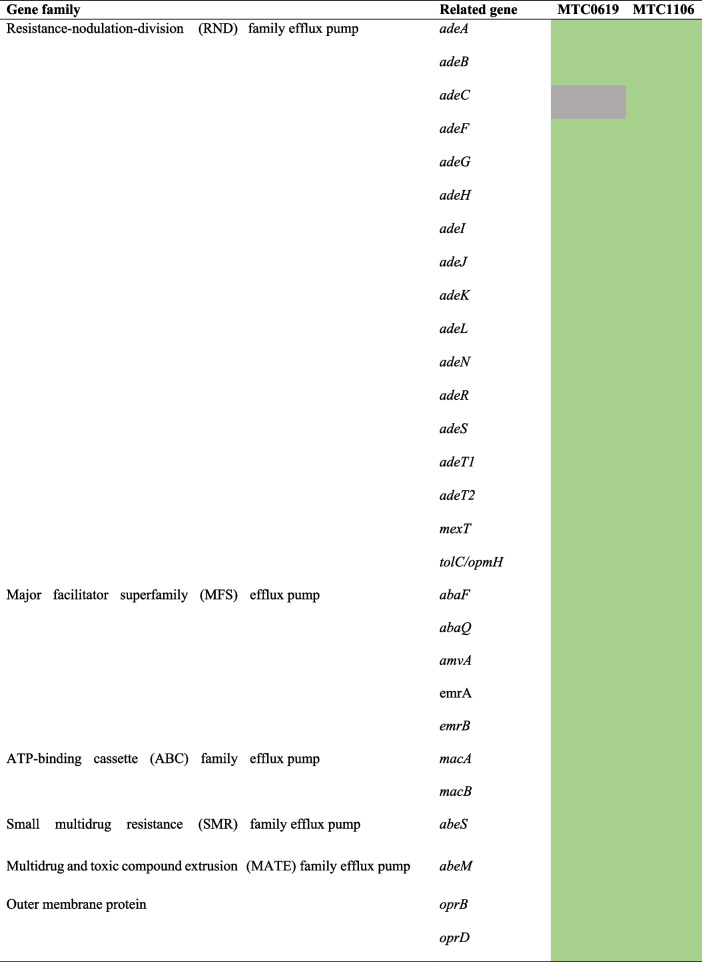
The list of genes encoding efflux pumps and outer membrane proteins related to AMR in MTC0619 and MTC1106

Remarks:

Gene absence

Gene presence

The occurrence of aminoglycoside resistance genes displayed some variations between the two isolates. In the case of MTC0619, five related genes were identified, all of which fell into three distinct groups of aminoglycosides modifying enzymes (AMEs): N-acetyltransferase (AAC), O-nucleotydyltransferase (ANT), and O-phosphotransferase (APH). On the other hand, isolate MTC1106 lacked the AAC genes found in MTC0619 but notably carried the *armA* gene, which encodes 16S rRNA methyltransferase, belonging to the category of aminoglycoside target-modifying enzymes.

In addition to acquired resistance genes, several chromosomal mutations associated with resistance to fluoroquinolone and colistin were observed in *A. baumannii* MTC0619 and MTC1106. Chromosomal non-synonymous single nucleotide polymorphisms (SNPs) related to fluroquinolone resistance were identified in the quinolone resistance determining region (QRDR), which includes the *gyrA* and *parC* genes. Both isolates had double mutation, *gyrA* S81L and *parC* S84L, which have been demonstrated to render considerable increase in the MIC of fluroquinolones among *A. baumannii* isolates compared to those with only single or without mutation [29, 30]. Another *parC* non-synonymous substitution (A661V) was identified in MTC0619. Both isolates possessed *gyrB* mutations but none of these mutations have been demonstrated to influence fluroquinolone MICs. Despite MTC0619 having more nucleotide substitution in these three related genes, its MIC for the two tested fluroquinolones were lower than MTC1106, and notably, it remained susceptible to levofloxacin.

Regarding the colistin resistance, both isolates displayed low-level of resistance based on the MIC value (4 mg/L). MTC1106 appeared to have relatively more mutated genes. In particular, the nucleotide substitutions observed in the *pmrB* gene of MTC1106 were responsible for alterations in two amino acid positions, but none of non-synonymous *pmrB* mutations were found in MTC0619. It is worth noting that MTC1106 also harbored a copy of *pmrC* homologue denoted as *eptA* (92.65% sequence identity to *pmrC*) encoded for lipid A phosphoethanolamine (PetN) transferase. Along with acquired resistance genes, the presence of efflux pumps and outer membrane proteins that are commonly associated with antimicrobial resistance were also obtained from CARD and MEGARes databases [26, 27]. According to the summary of the results in Table 3, both MTC0619 and MTC1106 exhibited almost similar number of identified genes encoding those proteins. The difference between the two isolates was only on the absence of *adeC* in MTC0619, which has been known as part of *adeABC* RND-type multidrug efflux pump.

### Predicted fragments of genomic island and plasmid harboring AMR genes

**Fig. 3:**
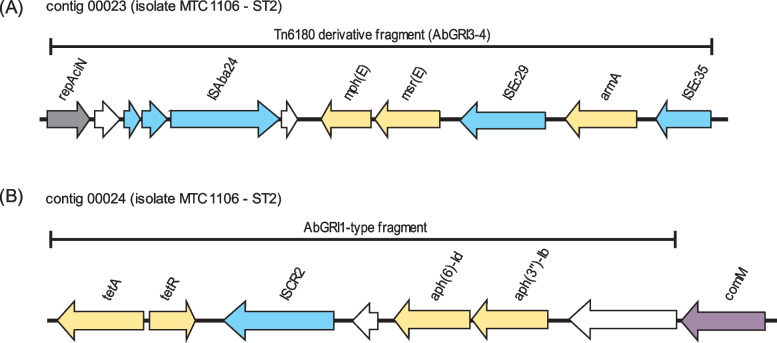
Schematic illustration of Tn6180-derived fragment of AbGRI3 (**A**) and AbGR11-type (**B**) fragment in MTC1106. The resistance genes and IS elements are shown in yellow and blue arrows, respectively. The uncolored arrows indicate hypothetical proteins while the *rep* gene of AbGRI3 dan *comM* gene of AbaR insertion target are depicted grey and purple arrows

In addition to the presence of IS elements, other types of mobile genetic elements were also identified within the assembled contigs. Both isolates MTC0619 and 1106 had seven prophage regions predicted in the contigs and most of them were considered incomplete (Table S1). None of AMR genes were notably found within the prophage regions identified in this study. A segment with similar structure to Tn6180-derived fragment of resistance island AbGRI3 in XDR *A. baumannii* SGH0908 [31] was present in MTC1106. This segment contained two genes associated with macrolides resistance genes, *mph(E)* and *msr(E)*, and also encompassed the genomic region where the *armA* gene is located (Fig. 3A). Another chromosomal resistance island identified in MTC1106 was the fragment of AbGRI1 which contained the two APH genes *aph(3”)-Ib* and *aph(6)-Id* (also known as *strA* and *strB*), along with *tetA(B)* tetracycline resistance determinant (Fig. 3B). This resistance island has notably been characterized among global clone 2 (GC2) strains of *A. baumannii*, and it employs *comM* gene as the target site of insertion, similar to the AbaR resistance island identified in GC1 isolates [32].

Based on the contig alignment against the *Acinetobacter* Plasmid Typing database [33], two *rep* genes encoding for replication initiation protein were detected in isolate MTC1106 (belonged to Rep types R3-T1 and R3-T60). The R3-T1 *rep* gene was situated in a small contig 00051 (8,842 bp). This contig also included BrnT/BrnA type II toxin-antitoxin system but did not contain any genes related to AMR. It is worth noting that the identification of R3-T60 presented an elusive finding since it was also identified as *repAciN*, a component of the AbGRI3-4 in contig 00023 (66,986 bp) mentioned earlier. However, the notion of this gene as part of plasmid replicon appeared less plausible due to the presence of chromosomal genes in the contig, such as tRNA elements and Csu pili biogenesis proteins. It has also been suggested that the AbGRI3-4 may actually be part of the chromosome as *repAciN* does not serve as a functional replication initiation protein [31].

**Fig. 4:**
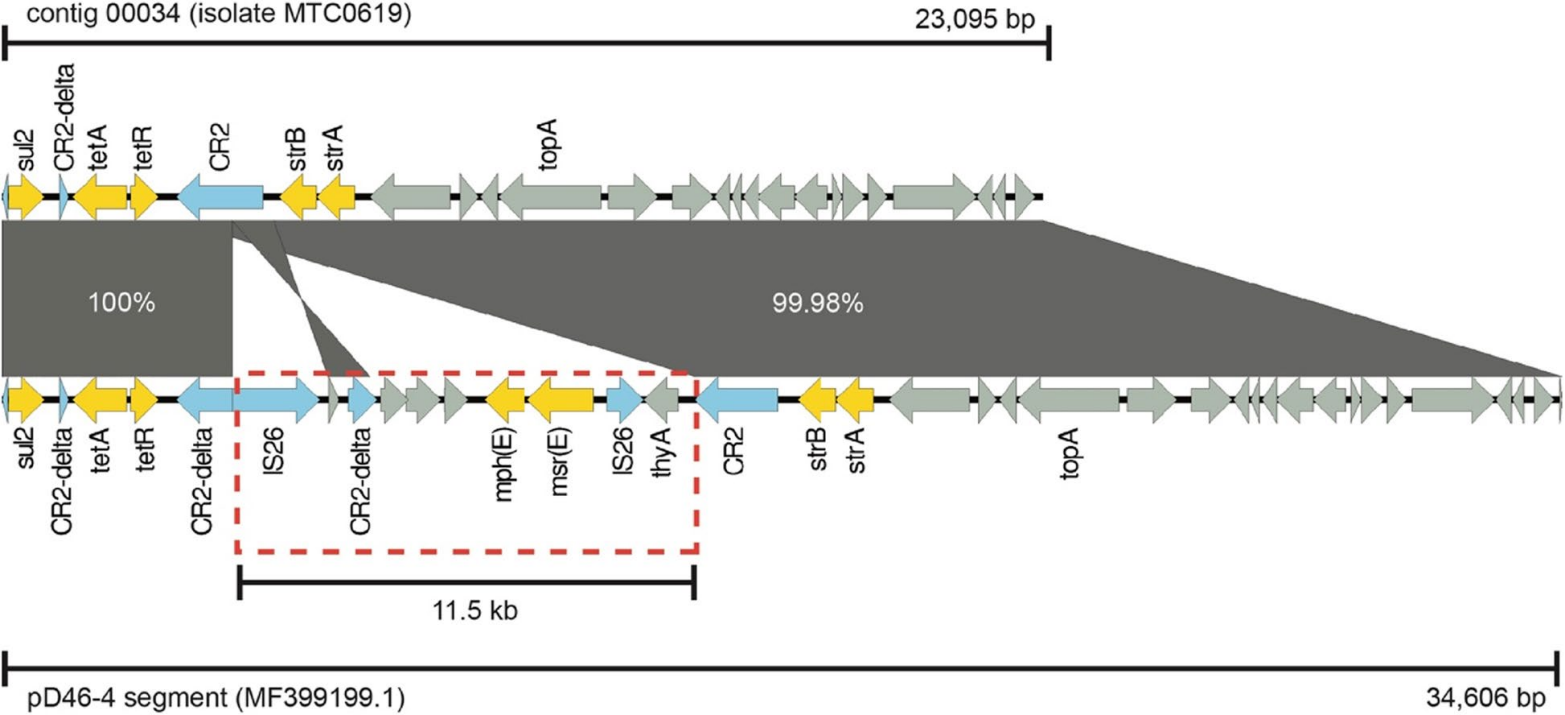
Linear comparison between a predicted plasmid contig in MTC0619 and its near-identical segment in pD46-4 (MF399199.1). The resistance genes and IS elements are in yellow and blue arrows respectively. A red-dotted box indicates the missing region of pD46-4 in MTC0619

Conversely, isolate MTC0619 did not exhibit any *rep* genes. The resistome profile of MTC0619 indicated the presence of genetic cassette comprising *tetA(B)-strA-strB* genes but not located adjacent to *comM*. Further investigation revealed that this segment is possibly a region of plasmid. Specifically, the sequence was identified within a contig spanning 23,095 bp, and a Blastn analysis revealed its similarity to a segment within a plasmid known as pD46-4 (GenBank Accession MF399199.1). This particular plasmid had been obtained from an ST-25 (Pasteur) XDR *A. baumannii* isolate and had been characterized as a large plasmid, encompassing 207,977 bp sequence [34]. Nonetheless, some differences were observed between this predicted plasmid contig and its corresponding near-identical segment in pD46-4. Specifically, a large region constituting around 11,500 bp of the Tn6183 in pD46-4 was noticeably absent in the plasmid fragment of isolate MTC0619 (Fig. 4).

### Pangenome analysis

**Fig. 5:**
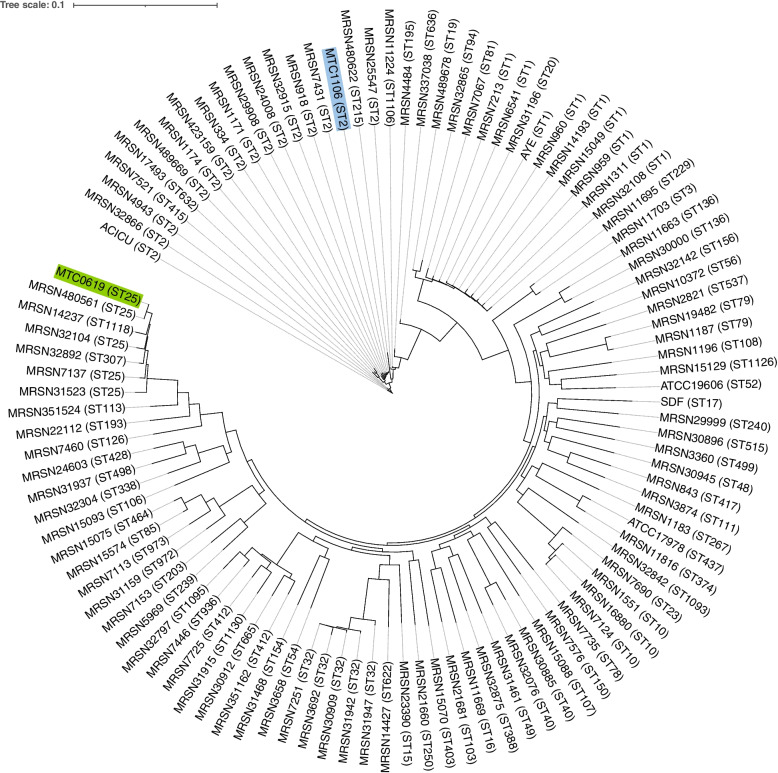
Phylogenetic tree based on the core genome SNPs from initial pangenome analysis involving two CRAB isolates in this study, five reference strains, and 100 publicly available genomes of diverse *A. baumannii* isolates. The isolates MTC0619 (ST25) and MTC1106 (ST2) were highlighted in green and blue, respectively

Given the well-documented genomic plasticity and clonal diversity of *A. baumannii*, the draft genomes of MTC0619 and MTC1106 were compared with a diverse panel of *A. baumannii* clinical isolates [35]. Five reference strains (ATCC 19606, ATCC 17978, ACICU, AYE, and SDF) were additionally included in the analysis to represent the model strains commonly employed in experimental studies (Table S2). A total of 19,125 gene clusters were identified, comprising 2,063 genes present in more than 99% genomes (core), 403 genes found in 95–99% of the genomes (soft-core), 1,906 genes present in15-95% of the genomes (shell), and 14,753 genes in less than 15% genomes (cloud) (Fig. S2). The majority of the genes classified within the accessory genome (77.69%) were annotated as hypothetical proteins. Additionally, a limited number of genes (< 50) associated with metal resistance and iron uptake regulation were observed in the accessory genome. The inference tree of accessory genomes (Fig. S3) indicated that isolate MTC0619 was more related to strain MRSN480561, consistent with the phylogenetic tree derived from the core genome SNPs (Fig. 5). On the other hand, isolate MTC1106 showed a different pattern of genetic relatedness. Its accessory genome content was more closely related to the strain MRSN17493 (ST632), a clinical isolate that has been characterized as a PDR strain [35]. However, phylogenetic tree of the core genome SNPs demonstrated that isolate MTC1106 exhibited a close genetic relatedness to an MDR strain MRSN7421 (ST2).

**Fig. 6:**
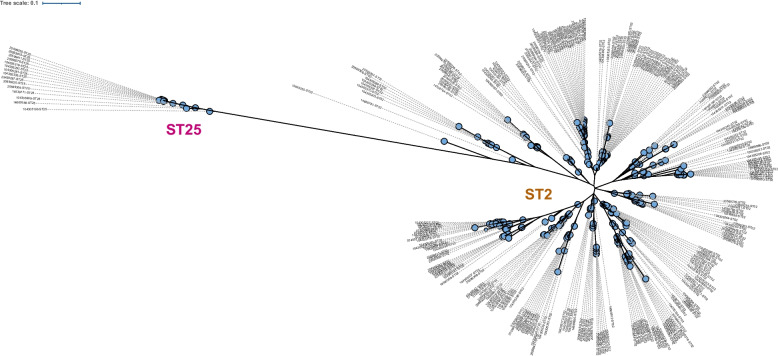
Inference tree of the Thai isolates belonging to ST2 and ST25 based on the presence and absence of gene clusters in pangenome analysis. The nodes are labelled based on the BioSample accession number and ST

Furthermore, the diversity of genomic content among *A. baumannii* belonging to ST2 and ST25 in Thailand was investigated. In this context, the two isolates sequenced in the present study were compared with publicly available genome data of Thai isolates. A total of 260 ST2 isolates and 14 ST25 isolates retrieved from previously published genomes [23] and the PathogenWatch database were included in this analysis (Table S3). The inclusion of these two specific STs in this study led to the construction of a pangenome comprising a total of 7,694 gene clusters, consisting of 2,722 (35.38%) core genes, 164 (2.13%) soft-core genes, 1,082 (14.06%) shell genes, and 3,726 (48.43%) cloud genes (Fig. S4). A clear distance between these two clones was indicated by the inference tree of accessory genome content (Fig. 6, Fig. S5). Notably, a consistent presence of genes encoding proteins associated with clustered regularly interspaced palindromic repeats (CRISPR) was observed among ST25 isolates (*n* = 15). These genes included *cas1*, *cas* 1, *cas3*, *csy1* (*cas8f1*), *csy2* (*cas5f1*), *csy3* (*cas7f1*), and *cas6f*. None of isolates in the ST2 lineage harbored *cas* genes.

### Gene expression analysis related to resistance phenotype

**Fig. 7:**
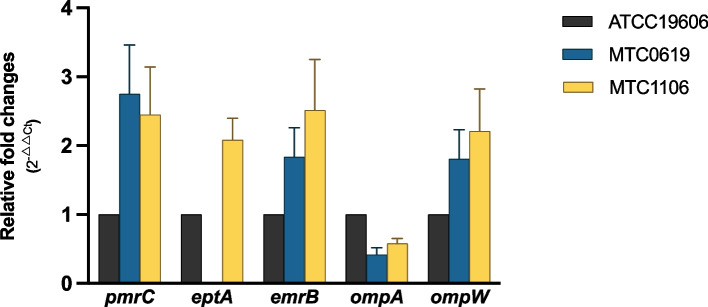
Expression of genes related to AMR phenotype in clinical *A. baumannii* MTC0619 and MTC1106 relative to the reference strain ATCC 19606. The expression levels were normalized to the housekeeping gene *rpoB*. The bars indicate the means from three independent experiments and the error bars represent the standard deviations of the means. The *eptA* was absent in MTC0619

The assessment of gene expression was conducted using quantitative real-time polymerase chain reaction (qRT-PCR) to further investigate the potential role of several antimicrobial resistance (AMR) genes identified in MTC0619 and MTC1106, particularly in conferring resistance to colistin. The results revealed that nearly all of the selected genes exhibited relatively higher transcript levels in comparison to expression in the calibrator strain (ATCC 19606), as shown in Fig. 7. Notably, the isolate MTC1106 displayed at least two-fold increase in the expression of genes encoding for PetN transferases (*pmrC* and *eptA*), the inner membrane transporter protein of the EmrAB efflux pump system (*emrB*), and the outer membrane protein W (*ompW*). In contrast to the upregulation observed in other genes, the expression of the outer membrane protein A (*ompA*) was notably lower in both isolates.

## Discussion

In this study, genetic characteristics of clinical carbapenem-resistant *A. baumannii* isolates from two main sequence types in Thailand, namely MTC0619 and MTC1106 were investigated and revealed some important genetic determinants such as antimicrobial resistance genes and mobile genetic elements, including fragment of resistance islands and plasmid. These isolates showed considerably high level of resistance to most tested agents within those nine antimicrobial categories Generally, MTC1106 appeared to harbor relatively more antimicrobial resistance genes than MTC0619 which in accordance with susceptibility profiles of the isolates. For example, the predicted ISAba1 region upstream of *bla*_OXA-23_ gene were related to carbapenem resistance since this IS element has been well-characterized to mediate overexpression of carbapenemase gene and eventually provide capability to confer resistance. Moreover, comparative genomics analysis was also helpful to identify the presence and absence of resistance genes in those isolates relative to several reference isolates and publicly available *A. baumannii* genomes. The results of genome annotation depicted higher number of genes predicted in RASTtk than Prokka annotation pipelines. Similarly, the RASTtk provided more functional annotation as evidenced by the number of identified hypothetical proteins. These two annotation pipelines identified the same number of rRNA and tRNA genes in both isolates. In the Oxford classification scheme, MTC1106 was classified into two different ST195 and 1816 because of the presence of two *gdhB* alleles (*gdhB_3* and *gdhB_189*) within its genome. The issue on *gdhB* duplication has been previously observed through a comparative evaluation of *A. baumannii* MLST schemes in which around 76% of tested *A. baumannii* strains were having duplicated *gdhB* gene [36]. Another study reported the failure to do Oxford-ST in one of *A. baumannii* strains owing to the presence of insertion sequence disrupting *gdhB* locus [37]. Collectively, some issues found in Oxford-MLST has been rising concern regarding its possibility to confuse the ST assignment of *A. baumannii*.

Molecular typing based on genetic loci involve in biosynthesis of capsular polysaccharides (K locus) and outer core of lipopolysaccharides (OC locus) using Kaptive database revealed that compared to other strains, less genes encoding proteins for capsule construction and processing were found in K locus of MTC0619 and its OC locus was shown to have more genes involving in complex sugar synthesis. The KL3 has been described as one of the most common KL types among *A. baumannii* strains, especially in ST2 and ST78. Meanwhile, the KL14 was shown to be the most prevalent in ST25 which is in accordance with the result in the present study [38]. The association of KL-OCL types with the virulence capability and other related phenotype of *A. baumannii* seems to remain elusive with relatively limited investigation has been done. However, an in vivo study using *Galleria mellonella* infection model reported the tendency of *A. baumannii* isolates with KL3 to exhibits lower virulence than those with KL14 locus [39]. This could be the basis to further evaluate the phenotype related to KL-OCL types of carbapenem-resistant *A. baumannii* isolates.

This study reported that the predicted ISAba1 and *bla*_*OXA-23*_ gene were separated by a 34-bp direct repeat in both isolates. The identified fragment of ISAba1 contained the IRL and promoter structure for expression of *bla*_*OXA-23*_, which closely resembled a previously documented finding. These characteristics were used to distinguish the presence of ISAba1 and ISAba4 in the upstream *bla*_*OXA-23*_ gene of *A. baumannii* isolates [40]. Specifically, it has been demonstrated that ISAba1 is located 34-bp upstream of the *bla*_*OXA-23*_, while ISAba4 is situated at 23-bp upstream of the gene. Both of those IS elements also displayed distinct promoter and inverted repeat sequences. Furthermore, a fragment of ISAba1 with a comparable promoter structure to a previous study was identified upstream of *bla*_*ADC-73*_ in isolate MTC1106. The insertion of this IS element in *bla*_*ADC*_ has been shown to mediate cephalosporins resistance in *A. baumannii* [41]. It is important to note that the short-read sequencing used in this study may not be sufficient to resolve the complex repetitive sequences of IS elements because the predicted ISAba1 sequences associated with *bla*_*OXA-23*_ and *bla*_*ADC*_ were found at the region of contig boundaries. Therefore, it remains inconclusive to exclude the possibility of other types of ISAba, as there could be a nearly identical genetic arrangement between IS elements or the co-insertion of different ISAba types. For instance, the ISAba9 was found to disrupt the ISAba1 insertion in *bla*_*OXA-51*_ and establishing hybrid promoter responsible for overexpression of the corresponding carbapenemase [42]. The insertion of ISAba125 in *bla*_*ADC*_ has been also reported to provide a different promoter orientation and is linked to a higher expression of the AmpC cephalosporinase [41]. The limitation of contiguity level from assembly using only short-read sequences may have also contributed to the identification of incomplete sequences of resistance islands and plasmid contigs in this study. Further investigations by incorporating long-read sequence data would help to address this issue as it allows to resolve fragmented contigs and may enable the assembly of finished genome.

Additionally, some chromosomal non-synonymous SNPs related to fluroquinolone resistance were identified in the quinolone resistance determining region (QRDR) comprising of *gyrA* and *parC* genes. Both isolates had double mutation in *gyrA* (S81L) and *parC* (S84L), a combination that has been shown to significantly elevate the MIC of fluoroquinolones in *A. baumannii* isolates when compared to isolates with a single mutation or those lacking these mutations [29, 30]. Another *parC* non-synonymous substitution (A661V) was identified in MTC0619. However, the prevalence of this mutation seems to be very rare with only one previous report describing it and there was no information regarding its significance on fluoroquinolone resistance [43]. The nucleotide substitution in *gyrB* have been also proposed to involve in fluoroquinolone resistance since the isolates with mutation in QRDR and several codons in *gyrB* were found to exhibit higher resistance to ciprofloxacin [29]. In the present study, both isolates had *gyrB* mutations but none of them have been demonstrated to influence the fluoroquinolone MICs. Although MTC0619 had more nucleotide substitution in those three related genes, the MIC of two tested fluoroquinolones was lower than MTC1106 and notably it was still considered susceptible to levofloxacin. Additionally, disruption of the *mar* operon by ISAba1 has been demonstrated to involve in fluoroquinolone resistance [44], but the corresponding operon in both MTC0619 and MTC1106 were found remain intact.

Colistin resistance in *A. baumannii* primarily occurs through the phosphoethanolamine (PetN) modification of lipopolysaccharides (LPS) and the loss of LPS itself. These mechanisms typically result from mutations in the *pmrCAB* and *lpxACD* operons [45]. While several mutations were identified within the genes constituting these operons in the two CRAB isolates in this study, it is worth noting that most of the identified mutations have also been reported in colistin-susceptible isolates [46, 47]. In addition, MTC1106 carried a copy of *pmrC* homologue denoted as *eptA* (92.65% sequence identity to *pmrC*) encoding lipid A PetN transferase. This gene was flanked by a phage-related integrase, but none of IS elements were detected around it. Based on the relative expression of *pmrC* in both isolates and *eptA* in MTC1106, it can be inferred that neither mutations in the *pmrCAB* operon nor the presence of *eptA* directly contribute to the low-level of colistin resistance observed in the two isolates. Some studies have reported that specific mutations in *pmrB* or the insertion of IS elements in the upstream region of *eptA* are responsible for significant overproduction of PetN transferases, thereby promoting the development of colistin resistance in clinical isolates [48–50]. The EmrAB efflux pumps may potentially play a role in conferring nonsusceptibility to colistin in MTC0619 and MTC1106 since a relative higher expression of *emrB* was observed, similar to a previous report [51]. However, it is also important to note that gene expression analysis conducted in this study reflects the expression at basal level, as no specific growth conditions that may alter the transcriptomic profile were introduced.

In terms of efflux pump genes, the differences between two isolates were only on the absence of *adeC* in MTC0619, which was known as a part of *adeABC* RND-type multidrug efflux pump. However, the lack of *adeC* has been suggested to be common among *A. baumannii* with the estimation of only 35% isolates carrying this gene [52]. Moreover, investigation on the functionality of AdeABC indicated that expression of AdeC was not essential due to insignificant difference of resistance profile displayed between mutant with *adeC* deletion and the parental strain harboring intact *adeABC* [53].

Data from pangenome analysis revealed a close relationship between MTC1106 and MRSN17493 which were defined as PDR strain according to the previous report [35]. The strains MRSN17493 was classified as ST632 in the Pasteur MLST scheme in which this ST was actually a single locus variant of ST2 whom MTC1106 was assigned to. Moreover, all of the AMR associated genes in strain MRSN17493, except *aph(3’)*, were also identified in MTC1106. On the other hand, MTC0619 was more related to strain MRSN480561. Both of these isolates belonged to the same ST25 and were considered XDR strains, albeit with distinct resistome profiles. The MTC0619 harbored less AMR genes and was non-susceptible to lower number of antimicrobials than the MRSN480561 strain. For example, the acquired macrolide resistance genes were absent in the MTC0619 but MRSN480561 harbored two types of this gene. The *bla*_*PER*_ gene was also not identified in MTC0619. However, this isolate already demonstrated to be colistin non-susceptible while the strain MRSN480561 was reported susceptible to colistin.

In the analysis comparing the two CRAB isolates in this study and a total of 274 publicly available genomes of Thai isolates, a smaller number of gene clusters constituting the pangenome was observed. The core genome was particularly larger compared to the analysis involving a diverse panel of isolates representing wide range of lineages, but the number of genes classified to accessory genome was lower. This distinct proportion of pangenome structure was due to only ST2 and ST25 employed in the later analysis. Interestingly, all of the ST25 isolates possessed the genes encoding for CRISPR effector proteins, suggesting the conservation of CRISPR-Cas system among Thai isolates belonging to this lineage. According to the recent classification scheme [54], the cluster of *cas* genes in these strains belonged to the type I-F CRISPR-Cas system. The presence of this bacterial defense system may influence the population dynamics of ST25 since it can limit the gene flow mediated by mobile genetic elements [55, 56]. Accordingly, the Thai isolates classified to ST25 clone exhibited a lower degree of intra-clonal diversity compared to the widely distributed ST2 as shown in the inference tree of accessory genome. In a previous study, some *A. baumannii* isolates harboring CRISPR-Cas system have been demonstrated to contain a lower number of resistance genes than the isolates without this system [57]. The presence of CRISPR-Cas systems has been also associated with a lower number of plasmids among *A. baumannii* isolates [58]. However, a contig representing a plasmid fragment was identified in the isolate MTC0619. The occurrence of plasmid among *A. baumannii* strains belonging to ST25 is relatively common based on some previous studies [34, 59, 60]. Hence, the role of this genetic determinant in the genome flux among strains in the ST25 lineage remains unclear, especially in regard to the acquisition of genetic determinants that drive the emergence of AMR phenotypes.

The advent of next-generation sequencing (NGS) technologies and genomic analysis pipelines has allowed whole-genome sequencing (WGS) which contributes to the expanding number of bacterial genome data from a variety of isolates representing broad genetic traits. One valuable data set generated from sequencing-based methods is the resistome profile, which consists of comprehensive AMR genes harbored in a bacterial genome. Comparative genomics analysis using NGS data, like the pangenomic-based approach, is also becoming feasible for a deeper understanding of resistance mechanisms and may eventually lead to the identification of molecular targets that can be considered in developing AMR strategies.

## Conclusion

Global spread of drug-resistant *Acinetobacter baumannii* clones has been regarded as one of the major public health concerns due to its increasing resistance to almost all available antimicrobials, including those belonging to last resort agents. In particular, the emergence of *A. baumannii* with non-susceptibility to carbapenems has been attributable to development of XDR and PDR strains. Surveillance on antimicrobial susceptibility pattern is essential to monitor the trend of antimicrobial resistance among *A. baumannii* isolates. Additionally, investigating genomic plasticity of this organism has been useful to provide insights regarding its propensity to easily acquire resistance determinants during clonal expansion. Genetic characteristics of clinical carbapenem-resistant *A. baumannii* isolates, namely MTC0619 (ST25) and MTC1106 (ST2) were investigated and revealed some important genetic determinants conferring antimicrobial resistance genes, mobile genetic elements, and fragment of resistance islands. Compared to MTC0619, MTC1106 generally seemed to harbor higher antimicrobial resistance genes. Some of those genetic determinants were present in accordance with susceptibility profiles of the isolates. Moreover, comparative genomics analysis identified the presence and absence of resistance genes in those isolates relative to several reference isolates and publicly available *A. baumannii* genomes. All in all, this study was likely the first comparative genomic analysis of carbapenem-resistant *A. baumannii* that specifically included the isolates belonging to ST2 and ST25 from Thailand. The results from this study showed the importance of susceptibility testing to monitor the resistance trend among *A. baumannii* isolates. The inadequate surveillance of CRAB strains may lead to emergence of XDR and PDR strains which is detrimental, especially for the healthcare system. Further investigations are still required to get insights about genotype and phenotype correlation, as well as the evolutionary trajectories of those carbapenem-resistant *A. baumannii*.

## Methods

### Bacterial collection and antimicrobial susceptibility testing

*A. baumannii* isolates were selected from a collection of clinical CRAB isolates obtained from 11 tertiary care hospitals across five regions in Thailand during the period of 2016 to 2017. The species identification of *A. baumannii* had been conducted through microbiological and biochemical testing, followed by genotypic confirmation for the presence of the *bla*_*OXA-51-like*_ gene and the MLST using a set of housekeeping genes to characterize strains by their unique allelic profiles.

The AST was performed manually in at least two independent experiments through either disc diffusion or broth microdilution according to the Clinical Laboratory Standards Institute (CLSI) guideline [61]. Prior to AST, a bacterial suspension in Mueller Hinton Broth (HiMedia Laboratories, India) with an OD_600_ of approximately 0.1 (equal to 0.5 McFarland standard) was prepared. Using a sterile cotton swab, bacterial inoculum was evenly spread onto 20-mL Mueller–Hinton agar (MHA) plates for the disk diffusion method. Antimicrobial disks (Oxoid, UK) were placed on the surface of the agar plate in triplicate and subsequently incubated overnight (16–18 h) at 37 °C. The zone of inhibition was measured by a digital caliper to determine susceptibility based on zone diameter breakpoints specific to each antimicrobial agent. In the broth microdilution method, the inoculum was added to 96-well plates along with a 2-fold dilution series of the tested antimicrobials in triplicates and then incubated overnight (16–18 h) at 37 °C. The MIC was determined by the lowest antimicrobial concentration at which no observable growth occurred, and the susceptibility categories were interpreted using the MIC breakpoints outlined in the CLSI guideline.

### Genomic DNA extraction and whole genome sequencing

The genomic DNA of the chosen isolates was extracted using the Gentra Puregene Yeast/Bact Kit (Qiagen, Germany) according to the manufacturer’s manual. The DNA concentration and purity (A260/280) were measured using a Nanodrop nucleic acid analyzer (Hercuvan Lab System, UK). Furthermore, the integrity and purity of the DNA samples were assessed by subjecting them to 1% agarose gel electrophoresis at 100 V for 40 min. The DNA samples underwent microbial whole-genome sequencing (WGS) using the Illumina Novaseq platform (Novogene Co. Ltd., China).

### De novo genome assembly

The paired-end reads obtained from WGS have been filtered in which the adapter sequence, reads with Phred quality scores (Q_Phred_) 5 or less, as well as reads with N greater than 10% were removed. To clean up the low-quality reads that might remain, a quality filtering step of the retrieved reads was done using fastp v.0.23.2 with the disabled adapter trimming function [62]. In addition, the raw reads were also checked for possible contamination by utilizing Kraken2 to assign taxonomic labels to the reads based on the miniKraken2_v2 database [63].

The reads that have been quality checked were then de novo assembled using Shovill v.1.10 (https://github.com/tseemann/shovill) with the SPAdes assembler to generate contigs with a minimum sequence length of 500 bp. The Shovill assembly pipeline by default allows pre-assembly processing steps of the reads, which include estimation of the genome size and read length using KMC3 [64], subsampling reads to achieve a sensible depth of 150X, error correction of the reads with Lighter [65], and merging overlapping reads with FLASh [66] to improve the quality of genome assembly. After SPAdes assembly, the Shovill pipeline also checks for assembly errors by mapping back the reads to contigs and subsequently doing error correction using Pilon [67] until the final contigs are eventually generated in FASTA format. Contigs were reordered using Mauve v.1.13 [68] with the Mauve contig mover (MCM) algorithm in Geneious Prime. The genome assembly of *A. baumannii* ATCC 17978 (NCBI Accession GCF_013372085.1, downloaded in January 2022) was used as the reference sequence. The ordered contigs for each isolate were annotated using Prokka v.1.14.6 and RASTtk. Furthermore, the quality of assembled contigs was evaluated using QUAST v.5.0.2 [69] and CheckM v.1.1.3 [70]. Functional classification was performed by inferring the predicted genes from Prokka to clusters of orthologous groups (COG) in the eggNOG-Mapper webserver [71]. In addition, predicted genes involved in metabolic pathways were analyzed by mapping them to the KEGG GENES database with bi-directional best hit method in the KEGG Automated Annotation Server (KAAS) [72].

### Molecular typing

Oxford scheme MLST of *A. baumannii* was performed using the MLST web server (https://cge.food.dtu.dk/services/MLST/) provided by the Center for Genomic Epidemiology with the contigs file as the input. The Pasteur scheme MLST was also carried out on the same web server to confirm the sequence type of the isolates. Additionally, Kaptive v.2.0.0 was employed to do capsular polysaccharide typing based on the *A. baumannii* K and OCL loci [38]. The FASTA files retrieved from Kaptive were utilized to create the annotated sequence of KL and OCL loci in Genbank format, which was then used to depict linear comparison with reference strains in Easyfig v.2.2.2 [73].

### Genome analysis for antimicrobial resistance genes and mobile genetic elements

Abricate v.1.0.1 (https://github.com/tseemann/abricate) in default parameters was used to do comprehensive screening of AMR genes with several databases, including ResFinder [25], NCBI AMRFinder Plus [26], CARD [27], and MEGARes [28]. The identified acquired AMR genes were further analyzed for the presence of associated mobile genetic elements using Mobile Element Finder web server (https://cge.food.dtu.dk/services/MobileElementFinder/) provided by the Center for Genomic Epidemiology, and manual screening from the annotated contigs based on IS elements identified from ISEScan [74]. Prophage regions were predicted using PHASTER [75]. Detection of the plasmid replicon was carried out using *Acinetobacter* Plasmid Typing scheme as previously described [33]. In addition, chromosomal point mutation associated with antimicrobial resistance were predicted by pairwise alignment in Geneious Prime using the sequence of related genes from isolates in this study and two drug-susceptible strains: *A. baumannii* ATCC 17978 (Genbank Accession CP053098.) and *A. baumannii* SDF (Genbank Accession CU468230.2).

### Comparative genomics and phylogenetic analysis

Roary v.3.12.0 [76] was used for pangenome-based comparative genomics analysis to get insight about genetic diversity of the CRAB isolates sequenced in this study. The assembled contigs of these isolates were particularly compared with a diverse publicly available genomes of *A. baumannii* clinical isolates described previously [35], along with five well-characterized *A. baumannii* strains (ATCC 19606, ATCC 17978, ACICU, AYE, and SDF) as shown in Table S2. A final dataset comprising 107 strains was prepared by reannotating the assembly file of each strain with Prokka v.1.14.6 and compiling the GFF files (.gff) as the input for pangenome analysis. The gene clusters were aligned with PRANK [77] in Roary. Subsequently, the roary plots script (https://github.com/sangerpathogens/Roary/tree/master/ contrib/roary_plots) was epmployed to generate visualization of the pangenome and Roary matrix against a tree based on the presence or absence of the gene clusters. Additional analysis was carried out in which the draft genomes of CRAB isolates in this study were compared with publicly available genomes of Thai isolates belonging to ST2 and ST25 isolates listed in a previous study [23] and PathogenWatch database (https://pathogen.watch/, accessed on August 15, 2023) The final dataset of this analysis comprised of 276 genomes as listed in Table S3. The core genome alignment file from Roary was used to extract SNPs of the core genome with SNP-sites v.2.5.1 [78]. The core genome SNPs phylogenetic tree was built in RaxML-NG v.1.1 [79] with General Time Reversible Gamma (GTR + G) evolutionary model and 1,000 bootstrap replicates. The phylogenetic tree was visualized in iTOL [80].

### Total RNA extraction and gene expression analysis

The bacterial isolates were cultivated in Luria Bertani (LB) broth (HiMedia Laboratories, India) until they reached the exponential growth phase, characterized by an optical density at 600 nm (OD_600_) ranging from 0.4 to 0.6. Subsequently, 2 mL of the culture was employed for total RNA extraction, following the FavorPrep™ Tissue Total RNA Mini Kit (Favorgen, Taiwan) manufacturer's instructions. RNase-free DNase I treatment (Qiagen, Germany) was carried out prior to elution to eliminate any potential DNA contamination. The concentration of the total RNA was determined spectrophotometrically using a Nanodrop nucleic acid analyzer. Approximately 5 µg of the RNA template was then reverse-transcribed into complementary DNA (cDNA) using the SuperScript™ IV First-Strand Synthesis System (Invitrogen, Lithuania).

For quantitative gene expression analysis, the cDNA was incorporated into KAPA SYBR FAST qPCR Master Mix (Kapa Biosystems, South Africa) in triplicate. The sequences of the primers utilized for amplification are presented in Table 4. The quantitative PCR (qPCR) was conducted using the Agilent Mx3000P qPCR system under the following conditions: an initial enzyme activation step for 5 min at 95 °C, followed by 50 cycles of denaturation at 95 °C for 10 s and amplification at 60 °C for 30 s. The relative expression of the target gene was determined by the comparative cycle threshold method (${2}^{-\Delta \Delta Ct}$). Notably, the *rpoB* gene was utilized for normalizing Ct values, with *A. baumannii* ATCC 19606 serving as the calibrator. Both the total RNA extraction and qPCR were carried out on three different occasions.

**Table 4:** List of primers used for RT-qPCR

Gene	Sequence	Reference
*rpoB*	F: 5’- GAGTCTAATGGCGGTGGTTC -3’ R: 5’- ATTGCTTCATCTGCTGGTTG -3’	[48]
*pmrC*	F: 5’- TGTTGCTACCTATTATGCGG -3’ R: 5’- ATTTTGAATTTGGTCGGGTG -3’	[48]
*eptA*	F: 5’- TCAGTTCTTTTCTTAGGGGC -3’ R: 5’- ATTTTGAATTTGGTCGGGTG -3’	[48]
*emrB*	F: 5’- GCGGGATGATTCCGACTTC -3’ R: 5’- TGAGCGTTTTGGTTCTGGAAA -3’	[81]
*ompA*	F: 5’- GGTATTCAGATAATTTTTCAGCAACTT -3’ R: 5’- AACAAATCAAACATCAAAGACCAA -3’	[82]
*ompW*	F: 5’- GCCTTATTTGCTCTGCCAAC -3’ R: 5’- CGTTTGAAACCATCACCATCT -3’	[82]

### Supplementary Information

**Additional file 1: Fig. S1. **Linear comparison of gene arrangement within (A) the K locus and (B) the OC locus between CRAB and five reference *A. baumannii* strains. **Fig. S2.** The pangenome structure of two CRAB isolates compared with 100 diverse clinical isolates and five reference strains (listed in Table S2). **Fig. S3.** Roary matrix and inference tree based on gene presence and absence among 107 genomes of *A. baumannii* isolates. **Fig. S4. **The pangenome structure of two CRAB isolates compared with other Thai isolates as listed in Table S3. **Fig. S5.** Roary matrix and inference tree based on gene presence and absence among 276 *A. baumannii* isolates of ST2 and ST25 from Thailand.

**Additional file 2: Table S1.** Predicted prophage regions in MTC0619 and MTC1106. **Table S2.** List of *A. baumannii* genomes included for pangenome analysis involving a diverse *A. baumannii *clones. **Table S3.** List of* A. baumannii* genomes included for pangenome analysis of Thai isolates belonging to ST2 and ST25.

## Data Availability

The datasets generated and/or analyzed during the current study are available from the DDBJ/ENA/GenBank under the BioProject accession PRJNA854605 with BioSample accession SAMN29439287 (Genome accession: JAMZNM000000000) and SAMN29439938 (Genome accession: JAMZNL000000000).
