# Supplementary material for: Comparative genotypic characterization related to antibiotic resistance phenotypes of clinical carbapenem-resistant Acinetobacter baumannii MTC1106 (ST2) and MTC0619 (ST25)
Source: BMC Genomics. 2023 Nov 17;24:689. doi: 10.1186/s12864-023-09734-2 (PMC10655397; doi:10.1186/s12864-023-09734-2)
Supplement: Supplementary file 1 — Additional file 1: Fig. S1. Linear comparison of gene arrangement within (A) the K locus and (B) the OC locus between CRAB and five reference A. baumannii strains. Fig. S2. The pangenome structure of two CRAB isolates compared with 100 diverse clinical isolates and five reference strains (listed in Table S2). Fig. S3. Roary matrix and inference tree based on gene presence and absence among 107 genomes of A. baumannii isolates. Fig. S4. The pangenome structure of two CRAB isolates compared with other Thai isolates as listed in Table S3. Fig. S5. Roary matrix and inference tree based on gene presence and absence among 276 A. baumannii isolates of ST2 and ST25 from Thailand. [file 12864_2023_9734_MOESM1_ESM.docx]

**
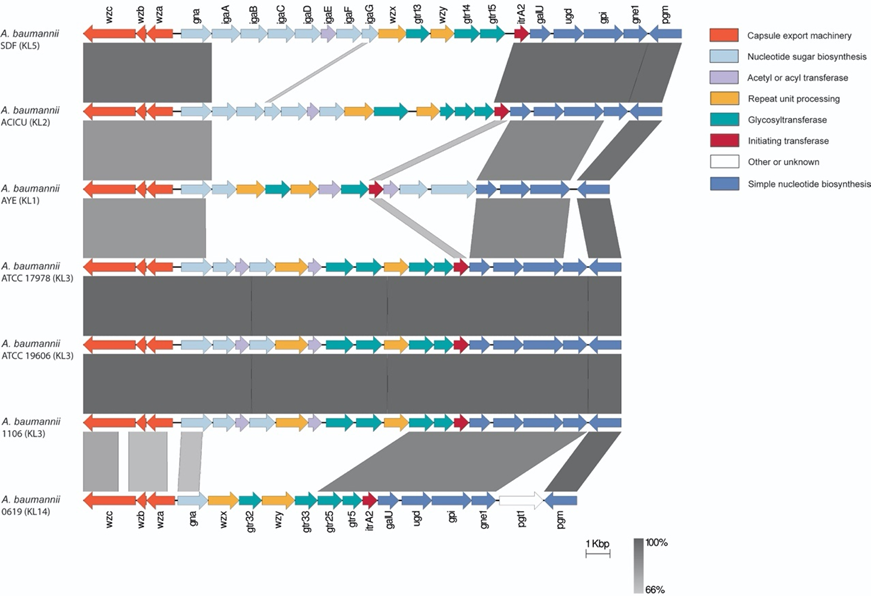
**

**(A)**

**
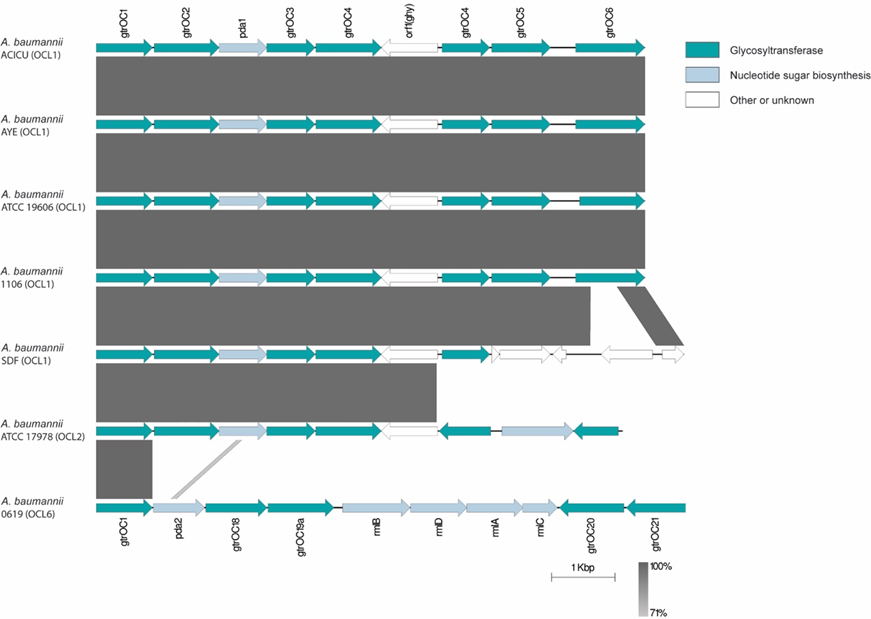
**

**(B)**

**Fig. S1** Linear comparison of gene arrangement within (A) the K locus and (B) OC locus between CRAB isolates and five reference *A. baumannii* strains. The genes are highlighted in different colors based on its function in the biosynthesis of K unit capsular polysaccharides and O-antigen polysaccharides.

**
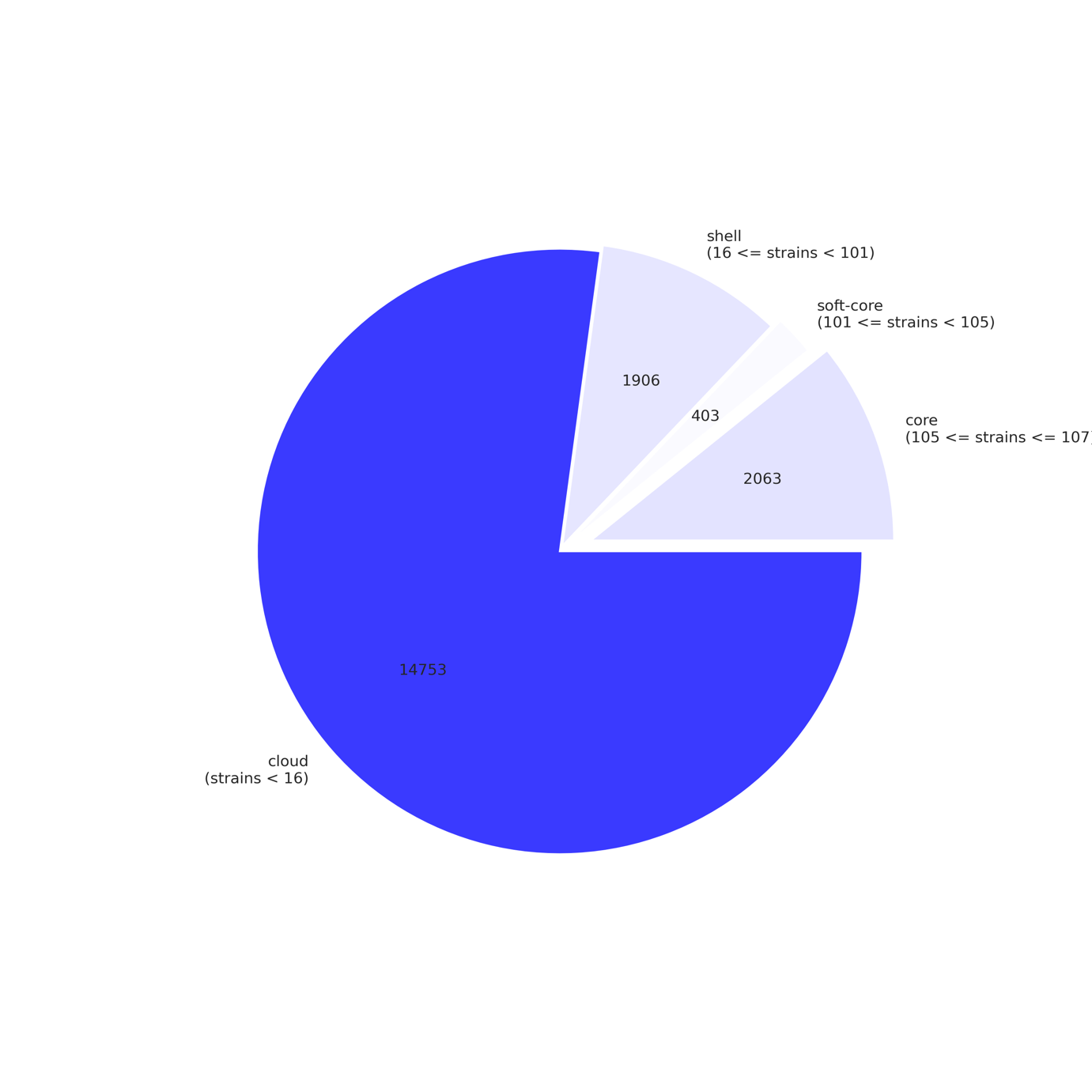
**

**Fig. S2** The pangenome structure of two carbapenem-resistant *A. baumannii* isolates compared with 100 diverse clinical isolates and five reference strains (listed in Table S2).

**
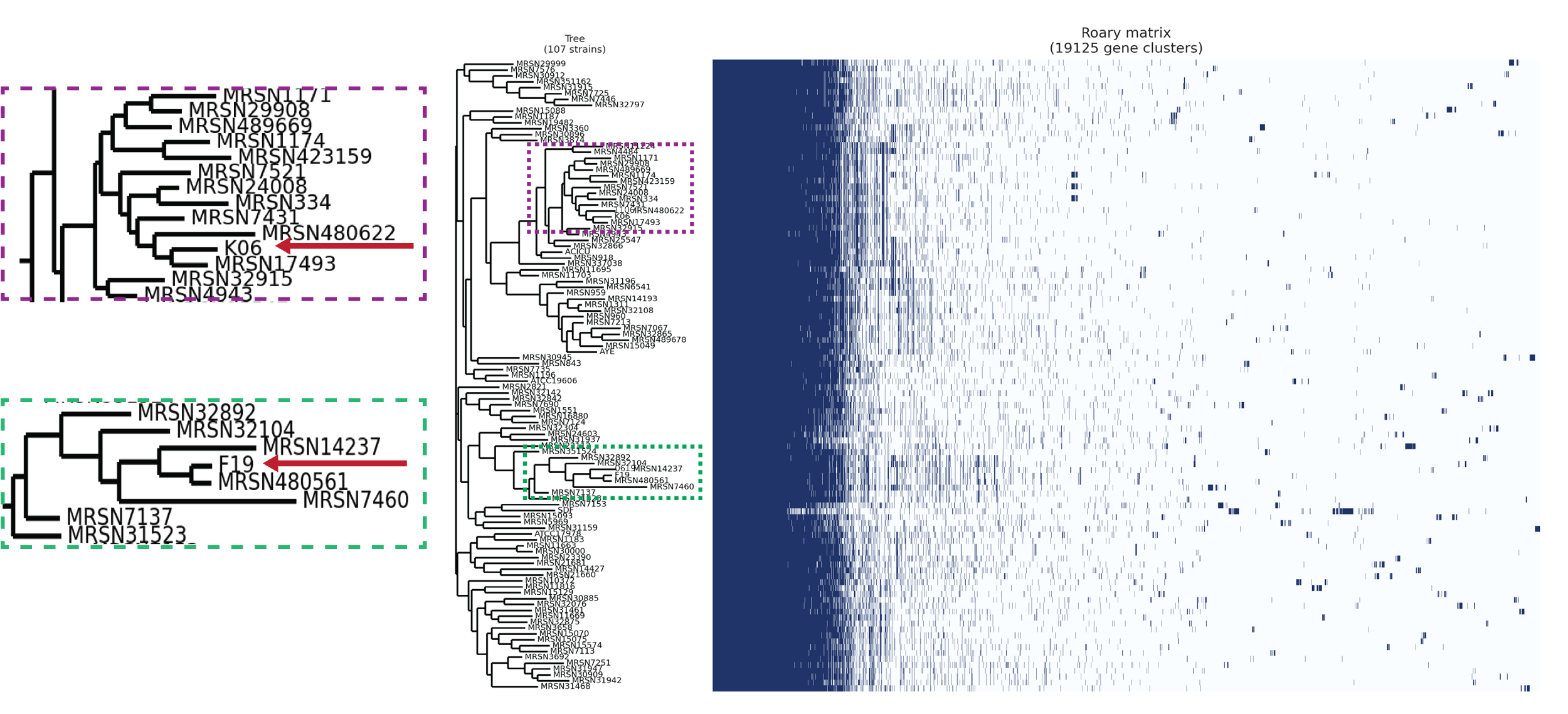
**

**Fig. S3** Roary matrix and inference tree based on gene presence and absence among 107 genomes of *A. baumannii* isolates. A zoomed overview showing MTC0619 (labelled as F19) and MTC1106 (labelled as K06) is provided in dotted-purple and dotted-green box, respectively.


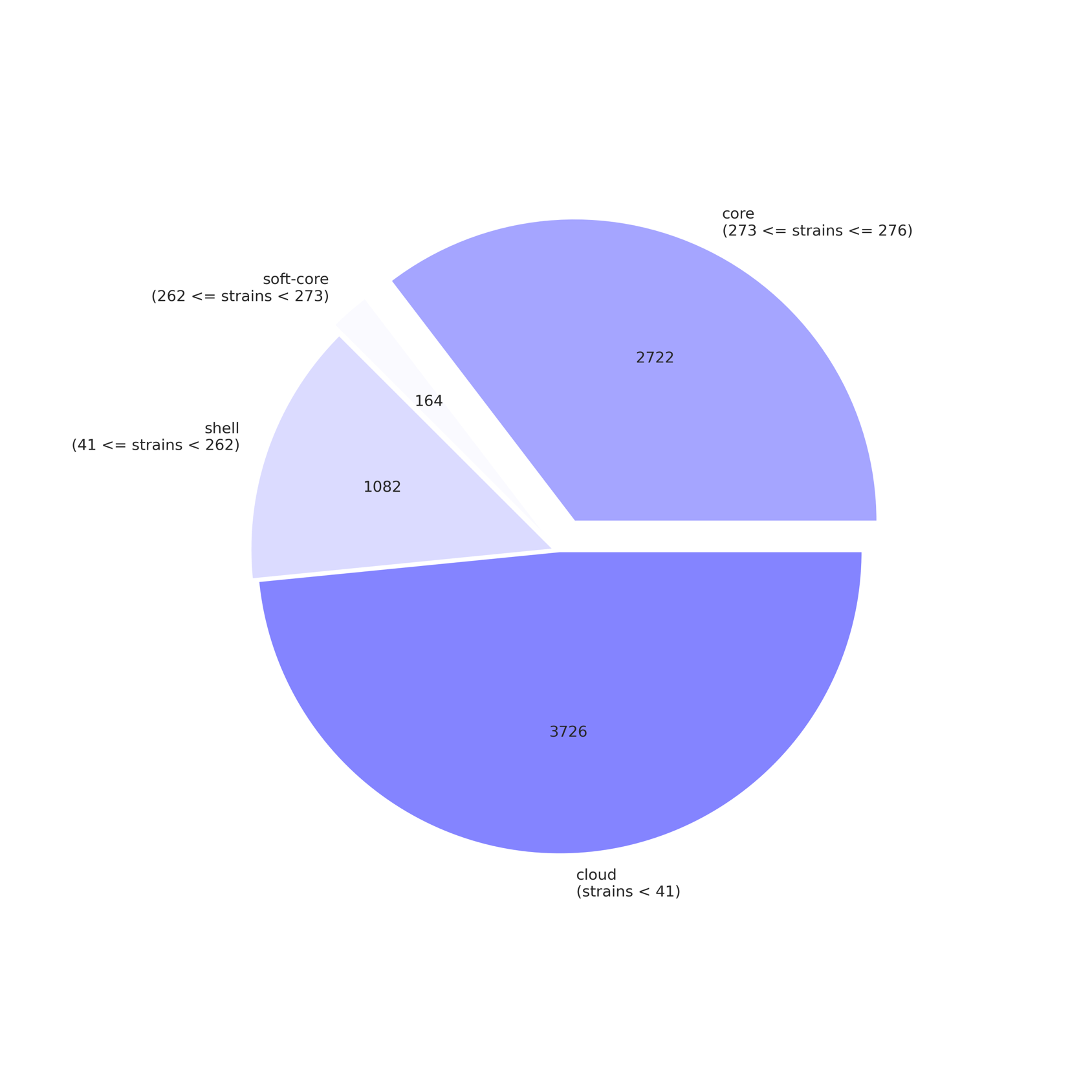


**Fig. S4** The pangenome structure of two carbapenem-resistant *A. baumannii* isolates compared with other Thai isolates. The dataset included 274 publicly available genomes belonging to ST2 (n=260) and ST25 (n=14) as listed in Table S3.


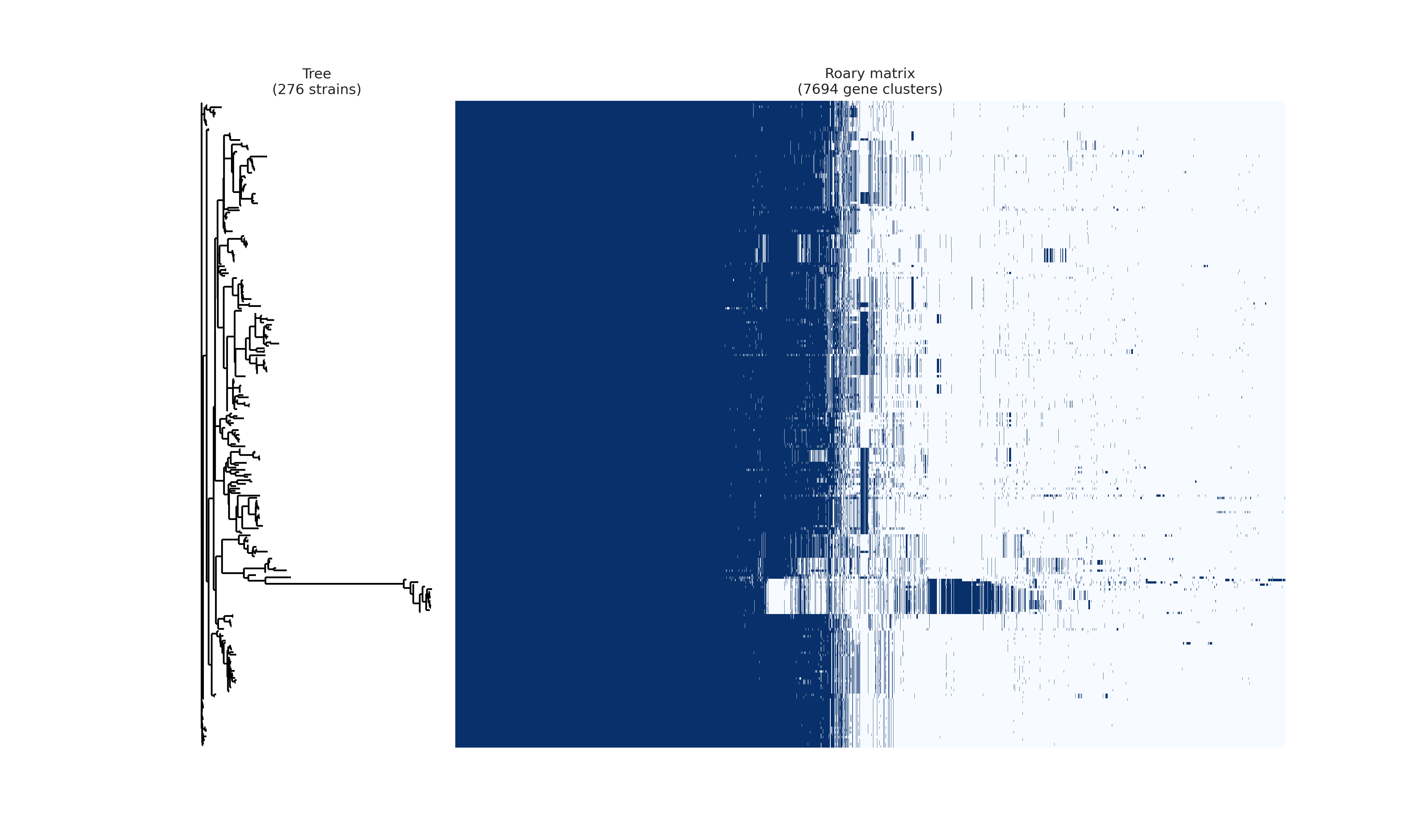


**Fig. S5** Roary matrix and inference tree based on gene presence and absence among 276 *A. baumannii* isolates of ST2 and ST25 from Thailand.
